# Study of Vitamin D Level and Vitamin D Receptor Polymorphism in Hypothyroid Egyptian Patients

**DOI:** 10.1155/2019/3583250

**Published:** 2019-08-26

**Authors:** Hoda A. ElRawi, Nashwa S. Ghanem, Naglaa M. ElSayed, Hala M. Ali, Laila A. Rashed, Mai M. Mansour

**Affiliations:** ^1^Int Med Dep Faculty of Medicine, Cairo University, Cairo, Egypt; ^2^Med Biochemestry Dep Faculty of Medicine, Cairo University, Cairo, Egypt

## Abstract

**Purpose:**

The current study aimed at assessing vitamin D level and vitamin D receptor polymorphism in hypothyroid Egyptian patients and its effect on hypothyroidism and thyroid morphology, also to find a causal relation between vitamin D and hypothyroidism.

**Methods:**

This case-control study was conducted on 35 hypothyroid patients and 35 matched unrelated healthy controls. Total serum 25-hydroxyvitamin D3 and thyroid antibodies were measured using a human ELISA kit. Genotyping was performed by using real-time PCR. HOMA-IR was also calculated (fasting insulin in mIU/L × fasting glucose in mg/dL/405). All subjects were assessed for thyroid morphology by thyroid ultrasonography.

**Results:**

Vitamin D level was lower in hypothyroid patients than in control subjects. Vitamin D was also inversely related to TSH, HOMA-IR, and levels of anti-TG and anti-TPO. VDR polymorphism (Fok1 and Apa1) had no relation to TSH or vitamin D levels in both patients and control groups. Low vitamin D levels were associated with increased thyroid vascularity and nodularity; furthermore, vitamin D was inversely proportional to thyroid gland volume. Correlation of HOMA-IR with the levels of both anti-TG and anti-TPO in the 70 subjects proved that HOMA-IR was positively correlated to both antibodies.

**Conclusion:**

This study confirmed the association of vitamin D deficiency with hypothyroidism, thyroid autoimmunity, increased volume, nodularity, and vascularity of thyroid gland in hypothyroid patients as well as increased HOMA-IR. It proved the association between HOMA-IR and thyroid autoimmunity. The study proved no association between VDR polymorphisms (Fok1 and Apa1) with either vitamin D levels or TSH levels.

## 1. Introduction

Vitamin D deficiency is a global health problem. Over a billion people worldwide have vitamin D deficiency or insufficiency [1]. The association between vitamin D deficiency and autoimmune diseases like rheumatoid arthritis, systemic lupus erythematosus, multiple sclerosis, and inflammatory bowel disease was described, and vitamin D supplementation prevents the development of these autoimmune diseases [1, 2].

The involvement of vitamin D in autoimmune thyroid disorders “AITD” has been of interest. Apart from its role in skeletal metabolism, vitamin D has been recognized as both an exogenous and an endogenous player in endocrinopathies such as type 1 and type 2 diabetes mellitus, adrenal diseases, and polycystic ovary syndrome [3, 4].

It is unclear whether low vitamin D levels are closely associated with the development of autoimmune thyroid disease. Some case-control studies have suggested that lower serum vitamin D levels or a higher prevalence of vitamin D insufficiency existed in patients with AITDs compared with that in healthy controls [3, 5]. Another study reported no significant association between serum vitamin D levels and thyroid autoimmunity [4].

However, a later study showed that low vitamin D levels have been associated with thyroid disease, such as Hashimoto's thyroiditis and new-onset Graves' disease. Impaired vitamin D signaling has been reported to encourage development of thyroid tumors [6].

Vitamin D has major biological activities including cellular proliferation and differentiation, immune system modulation, and muscle strengthening. An environmental factor may also be important in the etiology of T-cell-mediated autoimmune diseases. VDR gene contains more than 470 single nucleotide polymorphisms, which cause functional differences in immunomodulatory action of vitamin D. The most common polymorphisms of the VDR include Fok1 and Apa1 [7].

## 2. Aim of the Work

To clarify the relationship between vitamin D level, vitamin D receptor polymorphisms, hypothyroidism, serum thyroid autoantibodies, and HOMA-IR in the Egyptian hypothyroid patients.

### 2.1. Subjects and Methods

The current study is a cross-sectional case-control comparative study that was approved by the Ethical Committee, Internal Medicine Department, Kasr El Ainy Faculty of Medicine, Cairo University.

It was conducted on 70 Egyptian subjects that were divided into two groups.

#### 2.1.1. Group 1

This group included thirty-five newly discovered hypothyroid patients who did not receive L-thyroxine or received it for a period less than 6 weeks, with ages ranging from 21–52 years, 29 females (82.85%) and 6 males (17.14%). All patients were recruited from the endocrine outpatient clinic in Cairo University Hospitals (Kasr El Ainy Hospital).

#### 2.1.2. Group 2

This group included thirty-five age- and sex-matched apparently healthy volunteers serving as control group with ages ranging from 20–55 years, 28 females (80%) and 7 males (20%).

Diabetic patients, postmenopausal patients, patients having polycystic ovary syndrome, and hepatic impairment or renal impairment were excluded.

Written informed consent was obtained from all subjects before being enrolled in the study.

All cases and control subjects were subjected to complete history and clinical examination including weight, height, and BMI calculated as body weight in kg/height in m^2^ (Kg/m^2^).

Lab investigations including free T3, free T4, TSH, serum calcium (total and ionized), phosphorous, Mg, alkaline phosphatase (ALP), serum albumin, serum transaminases, urea and creatinine, parathyroid hormone level, 25 hydroxyvitamin D3 level, thyroid peroxidase antibody (anti-TPO) level, thyroglobulin antibody (anti-TG) level, HOMA-IR (fasting insulin × fasting glucose/405), vitamin D receptor polymorphism (Fok1 and Apa1), and thyroid gland ultrasound.

### 2.2. Kits Used in Biochemical and Molecular Assays

The following kits were used in the biochemical and molecular assays: vitamin D human ELISA kit, 96 tests/kit (Sunredbio, Shanghai, China); DNA extraction kit from whole blood, 100 tests/kit (Qiagen, USA); Taq man master mix for SNP detection, 100 tests/kit (Applied Biosystems, USA); anti-thyroid peroxidase human ELISA kit, 96 tests/kit (ThermoFisher Scientific, USA); and anti-thyroglobulin human ELISA kit, 96 tests/kit (Abcam, USA).

### 2.3. Details of Statistical Methods Used for Calculating the Results

Data were coded and entered using the statistical package IBM SPSS Statistics for Windows, Version 24.0., IBM Corp, Armonk, NY.

## 3. Results

All data of the subjects are presented in the following tables. A significant difference was detected on comparing BMI, total calcium, ionized calcium, PTH, phosphorous, ALP, fasting plasma glucose, fasting insulin, and HOMA-IR between the two studied groups while no significant difference could be detected on comparing magnesium between the two studied groups, as shown in Table 1.

**Figure 1:**
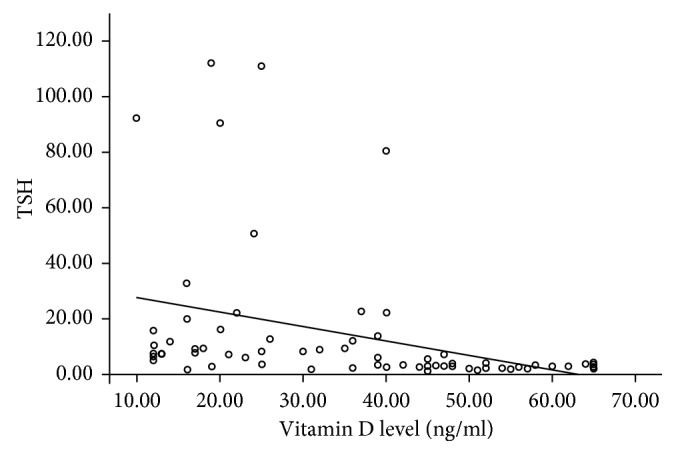
Relation of vitamin D levels with different TSH levels.

As regards vitamin D levels in our study, 45.7% (16 cases) of our patients showed vitamin D insufficiency, 22.8% (8 cases) were deficient, and 31.4% (11 cases) were having normal vitamin D levels. On the contrary, 5.7% (2) of our control subjects showed vitamin D insufficiency, 2.85% (1) were deficient, and 91.4% (32) were having normal vitamin D levels. This signifies that vitamin D insufficiency and deficiency are more frequent in patients in the hypothyroid group than in the control group. Vitamin D level was lower in hypothyroid patients than in control subjects (24.20 ± 10.78 versus 48.5 ± 12.57, *P* value is <0.001) (Table 1). There was significant negative correlation between vitamin D and TSH levels with *P* value <0.001 and *r* −0.600, as demonstrated in Figure 1.

Thyroid antibodies (anti-TPO and anti-TG) were measured in both control and hypothyroid groups. All patients in the hypothyroid group were found to have Hashimoto's thyroiditis. We found that anti-TG was higher in the hypothyroid group than in the control group. In addition, anti-TPO is higher in hypothyroid group than in control group. This variation is statistically significant as *P* value is <0.001 as shown in (Table 1).

There was statistically significant relation between vitamin D level in relation to thyroid antibodies in both the control group and hypothyroid group as *P* value is <0.001 (statistically significant). Vitamin D level was inversely proportional to levels of anti-TG and anti-TPO as correlation coefficients were −0.549 and −0.526, respectively.

Regarding VDR polymorphisms, two polymorphisms were studied, namely, Fok1 and Apa1. In Fok1, the FF genotype was present in 16 subjects (45.7%) in the control group and in 17 cases (48.6%) in the hypothyroid group, while Ff genotype was present in 15 subjects (42.9%) and 15 cases (42.9%) in the control group and hypothyroid group, respectively. The ff genotype was present in 4 subjects (11.4%) and 3 cases (8.6%) in the control group and hypothyroid group, respectively.

**Figure 2:**
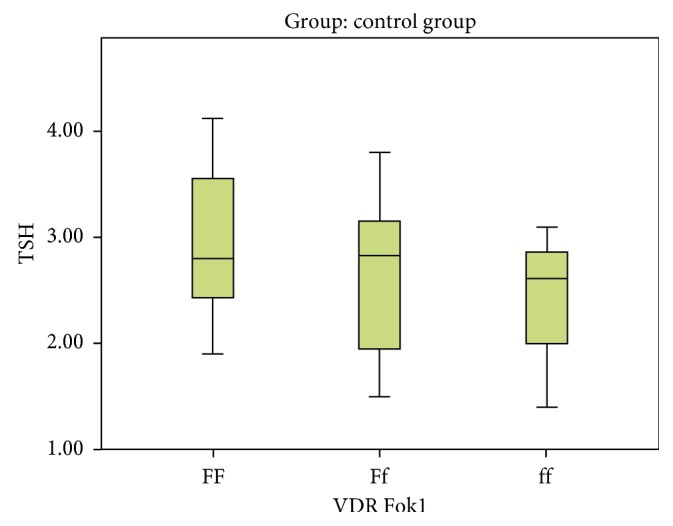
Variation of different Fok1 alleles with TSH levels in the control group.

**Figure 3:**
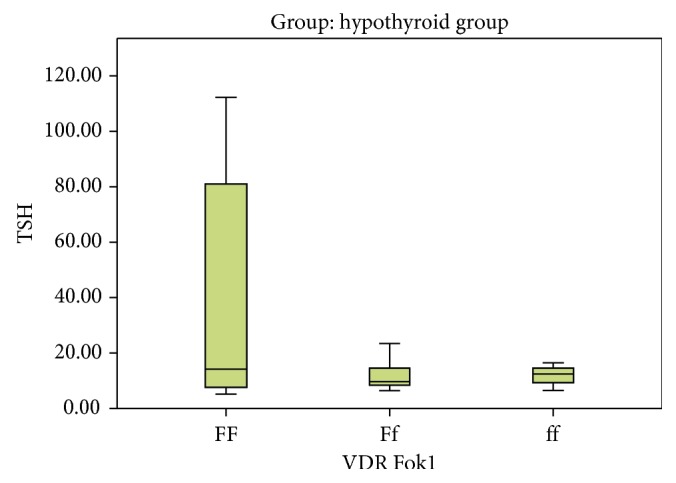
Variation of different Fok1 alleles with TSH level in the hypothyroid group.

On comparing Ff versus FF, no statistical significant association was detected ((*P*=0.904), OR = 0.941 with 95% CI (0.350–2.531)) between two studied groups. On comparing ff versus FF, no statistical significant association was detected ((*P*=0.678), OR = 0.706 with 95% CI (0.136–3.658)) between two studied groups (Table 2, Figures 2 and 3). Upon examining the allelic discrimination of Fok1, it was found that 67.1% of subjects carried the F allele (47 F alleles) and 32.9% of subjects carried the f allele (23 f alleles) within the control group and 70% of subjects carried the F allele (49 F alleles) and 30% of patients carried the f allele (21 f alleles) within the hypothyroid group. No statistical significant association was detected (*P*=0.716), OR = 0.876 with 95% CI (0.429–1.789), as shown in Table (2); this means that f allele has no relation to hypothyroidism.

**Figure 4:**
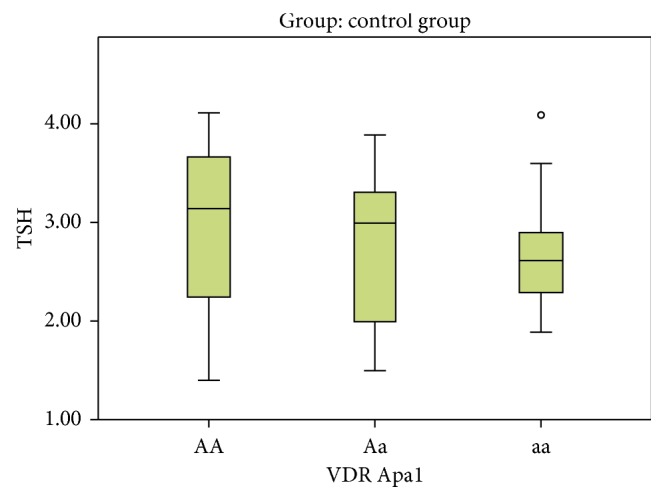
Variation of different Apa1 alleles with TSH level in the control group.

**Figure 5:**
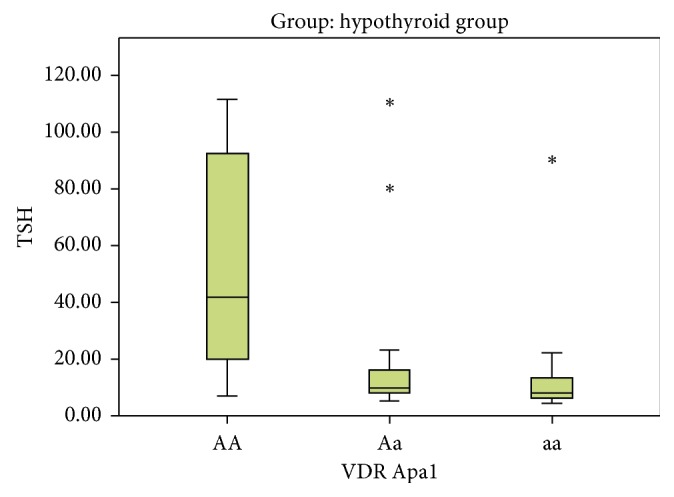
Variation of different Apa1 alleles with TSH level in the hypothyroid group.

In Apa1, the AA genotype was present in 4 subjects (11.4%) in the control group and in 6 cases (17.1%) in the hypothyroid group, while the Aa genotype was present in 13 subjects (37.1%) and 17 cases (48.6%) in the control group and hypothyroid group, respectively. The aa genotype was present in 18 subjects (51.4%) and 12 cases (34.3%) in the control and hypothyroid groups, respectively. On comparing Aa versus AA, no statistical significant association was detected ((*P*=0.854), OR = 0.872 with 95% CI (0.203–3.742)) between two studied groups. On comparing the aa versus AA, no statistical significant association was detected ((*P*=0.277), OR = 0.444 with 95% CI (0.103–1.915)) between two studied groups (Table 3, Figures 4 and 5).

Upon examining the allelic discrimination of Apa1, it was found that 30% of subjects carried the A allele (21 A alleles) and 70% of subjects carried the a allele (49 a alleles) within the control group and 41.4% of patients carried the A allele (29 A alleles) and 58.6% of patients carried the a allele (41 a alleles) within the hypothyroid group. No statistical significant association was detected ((*P*=0.160), OR = 0.606 with 95% CI (0.301–1.218)), as shown in Table 3; this means that a allele has no relation to hypothyroidism.

When comparing Fok1 alleles with vitamin D level in hypothyroid and control groups, we found that although the level of vitamin D varies between different alleles, this difference is insignificant (*P* value is 0.772 and 0.679, respectively) as shown in Table 4.

When comparing Apa1 alleles with vitamin D level in hypothyroid and control groups, we found that although the level of vitamin D varies between different alleles, this difference is insignificant (*P* value is 0.110 and 0.853, respectively) as shown in Table 5.

Thyroid ultrasound was done to both control and hypothyroid groups to compare presence of nodules, vascularity, and thyroid volume in relation to both vitamin D level and TSH values. Regarding thyroid volume, it was noted that thyroid volume in the control group was less than that in the hypothyroid group (mean 8.45 ± 2.62 SD and 13.47 ± 6.35 SD, respectively). This relation is statistically significant when *P* value is <0.001 as shown in Table 1.

Low vitamin D levels were associated with increased thyroid vascularity and nodularity (*P* value <0.001 and 0.038, respectively); furthermore, vitamin D was inversely proportional to thyroid gland volume as correlation coefficient was −0.458 and *P* value <0.001.

Regarding vascularity of thyroid gland, normal vascularity was found in those with mean vitamin D level 45.25 ± 16.32 SD, while increased vascularity was noted with lower vitamin D levels with mean 30.45 ± 14.67 SD. Thus, increased vascularity is associated with lower vitamin D levels, and this was statistically significant with *P* value <0.001. Also, normal vascularity was associated with lower TSH values with mean TSH 3.60 ± 2.86 SD if compared with those with increased vascularity in thyroid gland. Increased vascularity was associated with higher TSH values with mean 20.74 ± 30.25 SD. So increased vascularity was present in higher TSH values, and this was statistically significant with *P* value <0.001. Regarding free T3 and free T4, both were lower with increased vascularity, which was statistically significant as *P* value was 0.005 and 0.012, respectively, as shown in Table 6.

Nodules were found in those with mean vitamin D level 29.33 ± 15.07 SD, while absence of nodule were noted with higher vitamin D levels with mean 38.81 ± 16.94 SD.

Also, presence of nodules was associated with higher TSH values with mean TSH 16.38 ± 20.67 SD if compared with those with no nodules in thyroid gland. Absence of nodules was associated with lower TSH values with mean 13.02 ± 26.29 SD. So nodules were present in higher TSH values, and this was statistically significant with *P* value 0.003. Regarding free T3 and free T4, both did not differ with presence of nodules as *P* value was 0.093 and 0.903, respectively, as shown in Table 7.

**Figure 6:**
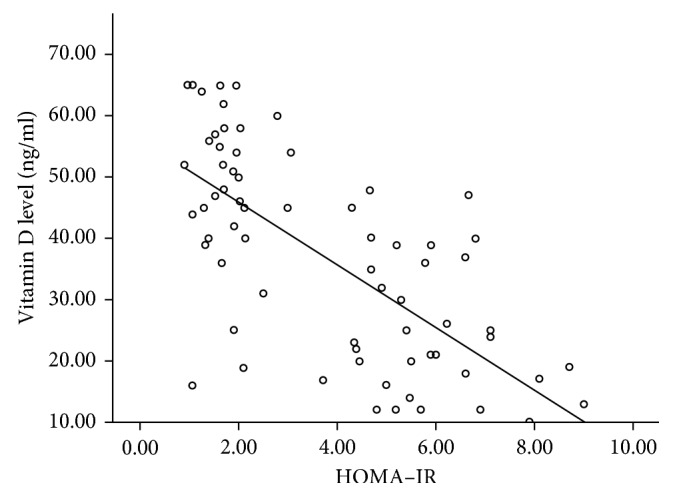
Relation between HOMA-IR and vitamin D.

When comparing HOMA-IR with vitamin D level in the 70 subjects, it was found that they are inversely proportional, which is statistically significant (*r* is ‒0.669 and *P* value is <0.001) (Figure 6 and Table 8).

Correlation of HOMA-IR with the levels of both anti-TG and anti-TPO in the 70 subjects proved that HOMA-IR was positively correlated to both antibodies (*r* 0.690 and 0.474, respectively, and *P* value in both is <0.001) (Table 8).

## 4. Discussion

This results of this study revealed that vitamin D is lower in the hypothyroid group than in the control group. Vitamin D insufficiency and deficiency are more frequent in patients in the hypothyroid group than in the control group. There is a significant negative correlation between vitamin D levels and TSH with *P* value <0.001.

Our study is supported by that of Bozkurt et al. which demonstrated that serum 25-hydroxyvitamin D (25OHD) levels of Hashimoto's thyroiditis (HT) patients were significantly lower than those of controls, and 25OHD deficiency severity correlated with the duration of HT [8]. Another study by Ke et al. also supports our study which found significant differences in serum 25OHD levels among mild HT, treated HT, and newly diagnosed (Graves' disease) GD patients (*P* < 0.001). Compared with the control, treated and mild HT patients exhibited significantly lower 25OHD levels (45.77 ± 3.48 vs. 83.49 ± 6.24 nmol/L, *P* < 0.001, and 55.25 ± 3.88 vs. 83.49 ± 6.24 nmol/L, *P* < 0.001, respectively) [9].

On the contrary to our study, Goswami et al., conducted a study on prevalence of vitamin D deficiency and its relationship with thyroid hormones and thyroid autoimmunity. They observed no significant difference between thyroid hormones in autoimmune thyroid disorders (AITD) and vitamin D levels [10]. Another two studies contradicted our findings; one is a case-control study conducted in the Kingdom of Saudi Arabia by Musa et al., 2017, which found an insignificant difference in the level of vitamin D among women with hypothyroidism compared to the control. The other is a cross-sectional study by Raposo et al., which was done in Portugal in 2017 and did not find any significant association or linear correlation between 25OHD levels or low vitamin D status with TSH, FT4, and FT3 levels after adjustment for sex and age. Difference between these studies and our study may be due to different sample sizes, different populations, and totally covered women (both control and hypothyroid were vitamin D deficient or insufficient) in the KSA study [11, 12].

We found that vitamin D was inversely related to levels of anti-TG and anti-TPO (*P* value <0.001 for both and correlation coefficient *r* −0.549 and −0.526, respectively). As regards to the role of vitamin D in autoimmune thyroid diseases, available data are still controversial, some authors support our results [13–15] but others do not [10, 16].

Our study demonstrated no relation between vitamin D receptor polymorphisms (Fok1 and Apa1) and vitamin D level or TSH level in both groups as *P* value was greater than 0.05. This is supported by a meta-analysis performed by Feng et al., who analyzed the associations between AITD risk and four polymorphisms (Bsm1, Fok1, Apa1, and Taq1) in the VDR gene and concluded that there was no significant association detected between FokI polymorphism and AITD risk [17]. The results of meta-analysis performed by Wang et al. suggests that the VDR-FokI polymorphism is associated with HT risk in overall population and in Asians, but not in Caucasians, and there is no association between TaqI, ApaI, and BsmI polymorphisms with HT risk neither in the overall population, nor in the ethnicity-stratified [18]. However, Djurovic et al. identiﬁed the association between VDR-FokI gene polymorphism and Hashimoto's thyroiditis in Serbian population as they found a significant difference in the genotype distribution of VDR-FokI polymorphism between patients with HT and controls (*P*=0.009) [19].

Our results showed that low vitamin D levels were associated with increased thyroid vascularity and nodularity (*P* value <0.001 and 0.038 respectively); furthermore, vitamin D was inversely proportional to thyroid gland volume as (correlation coefficient was −0.458 and *P* value <0.001) this is supported by previously mentioned studies [6, 8].

On the contrary to our findings, Ozdemir et al. studied serum vitamin D levels in patients with benign and malignant thyroid nodules. They found that serum vitamin D levels did not differ in patients with benign nodules and malignant nodules and patients without nodules [20].

In our study, HOMA-IR was significantly lower in the control group than in hypothyroid group (*P* value was <0.001). This is similar to a cross-sectional Egyptian study done by Hamdy et al. as their data showed that insulin resistance is significantly higher in the subclinical hypothyroid patient group than in the control group (*P*=0.05) [21]. The study of Sengupta et al. also supported our findings. It was done to assess HOMA-IR in mild newly discovered subclinical hypothyroid patients. They found that BMI, LDL-C, insulin levels, and HOMA-IR were elevated in the subclinical hypothyroid group [22].

In the current study, vitamin D was inversely related to HOMA-IR (r is −0.669 and *P* value is <0.001). Correlation of HOMA-IR with the levels of both anti-TG and anti-TPO in the 70 subjects proved that HOMA-IR was positively correlated with both antibodies (r 0.690 and 0.474, respectively, and *P* value in both is <0.001). This my suggest that insulin resistance could be a link between low vitamin D level and autoimmune hypothyroidism. The work of Varim et al. on insulin resistance in the patients with euthyroid HT supported our findings. Their results showed that high thyroid autoantibodies levels are related to high fasting blood glucose levels, insulin levels, lipid parameters, and HOMA-IR values [23]. Kavadar et al. studied the relationship between vitamin D status, physical activity, and insulin resistance in overweight and obese subjects, which supported our results that HOMA-IR is inversely proportional to vitamin D levels [24].

In our study, a significant difference was detected on comparing BMI, total calcium, ionized calcium, PTH, phosphorous, and ALP with *P* value <0.005 (statistically significant); however, magnesium does not show any difference between the control and hypothyroid groups with *P* value 0.108. Our results are consistent with those from a study by Mackawy et al., which was done to demonstrate association between vitamin D deficiency and its association with thyroid gland disease. They recorded a significant difference in serum calcium levels between the hypothyroid and control groups and significant positive correlations between serum 25(OH) vitamin D and serum calcium levels. On the contrary, they recorded significant negative correlations between serum 25(OH) vitamin D and TSH, with nonsignificant correlation with T4 [25].

## 5. Conclusion

This study confirmed the association of vitamin D deficiency with hypothyroidism, thyroid autoimmunity, increased volume, nodularity, and vascularity of thyroid gland in hypothyroid patients as well as increased HOMA-IR. It proved the association between HOMA-IR and thyroid autoimmunity. The study proved no association between VDR polymorphisms (Fok1 and Apa1) with either vitamin D levels or TSH levels. Further studies are recommended on a larger Egyptian population to speculate the link between low vitamin D, hypothyroidism, thyroid autoimmunity, and insulin resistance.

## Figures and Tables

**Table 1:** Clinical and laboratory data of the two studied groups.

	Group										
	Control group					Hypothyroid group					*P* value
	Mean	SD	Median	Minimum	Maximum	Mean	SD	Median	Minimum	Maximum	
BMI (kg/m^2^)	24.53	3.91	24.20	18.60	33.80	29.29	5.61	29.00	21.80	46.50	<0.001
Vitamin D level (ng/ml)	48.54	12.57	50.00	16.00	65.00	24.20	10.78	21.00	10.00	47.00	<0.001
Anti-TG (IU/ml)	51.69	31.39	41.00	24.00	150.00	147.69	50.46	147.00	32.00	246.00	<0.001
Anti-TPO (IU/ml)	25.69	21.15	18.00	10.00	108.00	58.97	32.85	54.00	18.00	121.00	<0.001
TSH (mIU/ml)	2.75	0.72	2.70	1.40	4.12	25.02	31.61	10.50	4.98	112.00	<0.001
FT3	2.91	0.62	2.80	1.50	5.00	2.13	0.89	1.90	0.20	3.90	<0.001
FT4 (ng/dl)	1.27	0.32	1.30	0.70	1.80	0.92	0.35	0.90	0.20	1.50	<0.001
PTH (pg/ml)	27.94	12.85	24.00	13.00	62.00	75.99	21.62	77.00	45.00	120.00	<0.001
Ionized Ca	1.26	0.05	1.27	1.14	1.30	1.09	.18	1.10	0.70	1.31	<0.001
Total Ca (mg/dl)	9.60	0.54	9.60	8.50	10.50	8.75	.91	8.70	6.50	10.30	<0.001
P	4.53	0.53	4.80	3.50	5.30	4.07	.53	4.10	3.00	5.40	0.001
Mg	2.21	0.43	2.20	1.60	3.50	2.41	.55	2.20	1.70	3.80	0.108
ALP	81.23	23.78	82.00	43.00	140.00	95.03	28.38	90.00	55.00	159.00	0.031
F. insulin (mIU/L)	9.46	3.44	9.00	4.00	22.00	27.06	4.87	26.00	18.00	40.00	<0.001
F. glucose (mg/dl)	80.86	8.64	82.00	58.00	99.00	88.71	9.85	89.00	71.00	107.00	0.001
HOMA-IR	1.84	0.72	1.69	0.90	4.67	5.89	1.29	5.70	3.70	9.00	<0.001
T. vol (mL)	8.47	2.62	8.03	4.44	19.19	13.47	6.34	11.15	4.09	25.90	<0.001

Data presented by mean ± SD, median, and absolute range. *P* value <0.05 is considered significant.

**Table 2:** Relation between alleles of Fok1 in both groups.

		Control group		Hypothyroid group		*P* value	OR	95% CI
		Count	(%)	Count	(%)			
VDR Fok1	FF	16	45.7	17	48.6	Reference		
	Ff	15	42.9	15	42.9	0.904	0.941	0.350–2.531
	ff	4	11.4	3	8.6	0.678	0.706	0.136–3.658
VDR Fokl1 alleles	Allele F	47	67.1	49	70.0	Reference		
	Allele f	23	32.9	21	30.0	0.716	0.876	0.429–1.789

*P* value <0.05 is considered significant. Data are represented by number and %.

**Table 3:** Relation between alleles of Apa1 in both groups.

		Control group		Hypothyroid group		*P* value	OR	95% CI
		Count	(%)	Count	(%)			
VDR Apa1	AA	4	11.4	6	17.1	Reference		
	Aa	13	37.1	17	48.6	0.854	0.872	0.203–3.742
	Aa	18	51.4	12	34.3	0.277	0.444	0.103–1.915
VDR Apa1 alleles	Allele A	21	30.0	29	41.4	Reference		
	Allele a	49	70.0	41	58.6	0.160	0.606	0.301–1.218

^*∗*^ *P* value <0.05 is considered significant. ^*∗∗*^Data are represented by number and %.

**Table 4:** Different Fok1 alleles in relation to vitamin D level in hypothyroid and control groups.

Vitamin D level (ng/ml)	VDR Fok1															
	FF					Ff					Ff					*P* value
	Mean	SD	Media	Minimum	Maximum	Mean	SD	Median	Minimum	Maximum	Mean	SD	Median	Minimum	Maximum	
Hypothyroid group	25.2	12.5	21.00	10.00	47.00	22.60	9.36	21.00	12.00	39.00	26.33	8.50	23.00	20.00	36.00	0.772
Control group	49.5	14.0	52.00	19.00	65.00	46.87	12.4	47.00	16.00	65.00	50.75	7.27	50.50	44.00	58.00	0.679

Data presented by mean ± SD. Data presented by median and absolute range. *P* value <0.05 is considered significant.

**Table 5:** Different Apa1 alleles in relation to vitamin D level in the hypothyroid group.

Vitamin D level (ng/ml)	VDR Apa1															
	AA					Aa					Aa					*P* value
	Mean	SD	Median	Minimum	Maximum	Mean	SD	Median	Minimum	Maximum	Mean	SD	Median	Minimum	Maximum	
Hypothyroid group	16.33	4.84	16.00	10.00	24.00	27.00	10.98	25.00	12.00	47.00	24.17	11.27	21.00	12.00	45.00	0.110
Control group	49.25	7.18	48.50	42.00	58.00	47.23	12.58	48.00	19.00	64.00	49.33	13.90	51.00	16.00	65.00	0.853

Data presented by mean ± SD. Data presented by median and absolute range. *P* value <0.05 is considered significant.

**Table 6:** Vascularity of thyroid gland in relation to vitamin D level and thyroid hormones.

	Vascularity										
	Normal					Increase					*P* value
	Mean	SD	Median	Minimum	Maximum	Mean	SD	Median	Minimum	Maximum	
Vitamin D level (ng/ml)	45.25	16.32	48.00	12.00	65.00	30.45	14.67	28.00	10.00	65.00	<0.001
TSH	3.60	2.86	2.90	1.40	15.69	20.74	30.25	8.23	1.79	112.00	<0.001
FT3	2.86	0.56	2.80	1.50	4.60	2.29	0.95	2.10	0.20	5.00	0.005
FT4	1.25	0.32	1.20	0.70	1.80	1.00	0.38	1.00	0.20	1.70	0.012

Data presented by mean ± SD. Data presented by median and absolute range. *P* value <0.05 is considered significant.

**Table 7:** Presence of nodules in thyroid gland in relation to vitamin D level, thyroid hormones, and HOMA-IR.

	Nodularity										
	Yes					No					*P* value
	Mean	SD	Median	Minimum	Maximum	Mean	SD	Median	Minimum	Maximum	
Vitamin D level (mg/ml)	29.33	15.07	28.00	10.00	58.00	38.81	16.94	40.00	12.00	65.00	0.038
TSH	16.38	20.67	9.90	2.46	92.50	13.02	26.29	3.55	1.40	112.00	0.003
FT3	2.24	1.14	2.02	0.20	5.00	2.62	0.72	2.70	1.00	4.60	0.093
FT4	1.09	0.34	1.10	0.40	1.80	1.10	0.39	1.10	0.20	1.80	0.903
Vitamin D level (mg/ml)	29.33	15.07	28.00	10.00	58.00	38.81	16.94	40.00	12.00	65.00	0.038
TSH	16.38	20.67	9.90	2.46	92.50	13.02	26.29	3.55	1.40	112.00	0.003
FT3	2.24	1.14	2.02	0.20	5.00	2.62	0.72	2.70	1.00	4.60	0.093
HOMA-IR	5.03	2.24	5.29	0.9	8.1	3.50	2.18	2.14	0.96	9	0.01

Data presented by mean ± SD. Data presented by median and absolute range. *P* value <0.05 is considered significant.

**Table 8:** Relation of HOMA-IR with vitamin D anti-TG and anti-TPO levels.

		Vitamin D level (ng/ml)	Anti-TG (IU/ml)	Anti-TPO (IU/ml)
HOMA-IR	*R*	‒0.669	0.690	0.474
	*P* value	<0.001	<0.001	<0.001
	*N*	70	70	70

*P* value <0.05 is considered significant.

## Data Availability

All data supporting the results are included within the article and are also available from the corresponding author.

## Additional Points

*Limitations*. The limitations of this study are the small number of subjects, limited ability to conclude that vitamin D status has direct relation to the pathogenesis of hypothyroidism, and that environmental factors, seasonal variations, personal activities, and exposure to sunlight were not taken into consideration in this study.
